# Cheerful People

**Published:** 1883-05

**Authors:** 


### CHEERFUL PEOPLE.

One melancholy person will mar the happiness of a household, particularly if he is an important member of the family. The father should not bring his business troubles home with him. Mothers have hundreds of little trials that fret the temper and cause the brow to cloud over ; but if it can possibly be helped such troubles should never be made apparent to the family circle. Of course it may be hard to suffer all to one's self, and it is so often a relief to pour into the ears of the assembled family the tribulations of the kitchen or nursery ; but this disposition is better checked in the beginning. The habit of complaining is one that grows unconsciously if indulged in at all. If an air of cheerfulness is assumed, however little it may be really felt, the spirit itself will soon pervade the soul, and each effort to subdue the fretful and gloomy feelings will be found easier.

No member of the family needs the buoyant influence of hope more than the wife and mother. Let her particularly cultivate this habit of mind if her husband is inclined to despondency. Her influence upon him is incalculable. But many a woman renders herself unfit for this duty by ruining her temper and nerves by overwork. That's a bad plan, mother. Don't try to do too much. Never mind if the baby's frock is unfinished ; put it by until another day. Don't be bent upon hanging up the fresh curtains to-day ; let them wait until you are not so tired. Don't work so hard as to unstring the nerves and make you incapable of proving yourself a cheerful companion to your husband and children in the evening. A brisk walk in the fresh air, a call on a pleasant neighbor, will give you something pleasant to think about, and supply you with items to enliven the tea table chat. You cannot afford to be melancholy, so drive away dull care and be cheerful, for your own sake as well as that of the household whose happiness depends upon your smile.

